# Associations of cardiorespiratory fitness, adiposity, and arterial stiffness with cognition in youth

**DOI:** 10.14814/phy2.14586

**Published:** 2020-09-19

**Authors:** Hannamari Skog, Niina Lintu, Henna L. Haapala, Eero A. Haapala

**Affiliations:** ^1^ Institute of Biomedicine School of Medicine University of Eastern Finland Kuopio Campus Finland; ^2^ Faculty of Sport and Health Sciences University of Jyväskylä Jyväskylä Finland

**Keywords:** adolescent, aerobic capacity, arterial health, body composition, cognitive functions, vascular stiffness

## Abstract

**Purpose:**

To investigate the associations of cardiorespiratory fitness, adiposity, and arterial stiffness with cognition in 16‐ to 19‐year‐old adolescents.

**Methods:**

Fifty four adolescents (35 girls; 19 boys) participated in the study. Peak oxygen uptake (V̇O_2peak_) and peak power output (W_max_) were measured by the maximal ramp test on a cycle ergometer and ventilatory threshold (VT) was determined with ventilation equivalents. Lean mass (LM) and body fat percentage (BF%) were measured using a bioelectrical impedance analysis. Aortic pulse wave velocity (PWVao) and augmentation index (AIx%) were measured by a non‐invasive oscillometric device. Working memory, short term memory, visual learning and memory, paired‐associate learning, attention, reaction time, and executive function were assessed by CogState tests.

**Results:**

V̇O_2peak_/LM (β = 0.36 *p* = .011) and W_max_/LM (β = 0.30 *p* = .020) were positively associated with working memory. W_max_/LM was also positively associated with visual learning (β = 0.37, *p* = .009). V̇O_2_ at VT/LM was positively associated with working memory (β = 0.30 *p* = .016), visual learning (β = 0.31 *p* = .026), and associated learning (β = −0.27 *p* = .040). V̇O_2_ at VT as % of V̇O_2peak_, BF%, PWVao, and AIx% were not associated with cognition.

**Conclusion:**

Cardiorespiratory fitness was related to better cognitive function, while BF% and arterial stiffness were not associated with cognition in adolescents.

## Graphical abstract

The evidence on the associations of cardiorespiratory fitness, adiposity, and arterial stiffness with cognition in adolescents is still limited and the evidence is mainly based on data from studies using indirect measures of fitness instead of directly measured peak oxygen uptake. Furthermore, there are no previous studies on the associations of the ventilatory threshold, a widely recognized submaximal measure of fitness, with cognition.

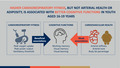

## 1 INTRODUCTION

The prevalence of overweight and obesity has increased (Broyles et al., 2010; Kautiainen, Rimpelä, Vikat, & Virtanen, 2002), while cardiorespiratory fitness (CRF) has declined among adolescents during the past decades (Tomkinson, Lang, & Tremblay, 2019). Adiposity and low CRF have been linked to an increased cardiometabolic risk in children and adolescents and an increasing body of evidence also suggests that they are associated with impaired cognition in youth (Donnelly et al., (2016); Hjorth et al., 2016; Trudeau & Shephard, 2008). Increased adiposity and low CRF have also been related to increased arterial stiffness, an early indicator of arteriosclerosis (McGill et al., (2000)). Furthermore, increased arterial stiffness has been associated with poorer cognitive functions in adolescents (Vogrin, Rupnik, and Micetic‐Turk (2017)) and older adults (Kramer, Erickson, & Colombe, 2006). However, the evidence on the associations of CRF, body fat content, and arterial stiffness with cognition in adolescents is still limited (Lamballais et al., 2018).

CRF, estimated by 20‐meter shuttle run test (Huang et al., 2015; Westfall et al., 2018) and peak oxygen uptake (V̇O_2peak_) scaled, that is, divided, by body mass (BM) have been positively associated with cognition in some (Chaddock, Erickson, Prakash, VanPatter, et al., 2010; Chaddock, Hillman, Buck, & Cohen, 2011; Chaddock, Hillman, et al., 2012; Davis et al., 2011; Voss et al., 2011), but not all studies (Chaddock, Erickson, Prakash, Kim, et al., 2010; Ruiz et al., 2010) in children and adolescents. However, scaling the measures of CRF by BM has no clear physiological rationale because it may not remove the effect of body size and composition on CRF (Tanner, 1949; Welsman & Armstrong, 2019). V̇O_2peak_ scaled by lean mass (LM) has been considered the most appropriate method to express CRF (Loftin, Sothern, Takashi, & Bonis, 2016). Furthermore, peak power output (W_max_) has often been considered a feasible indirect measure of maximal cardiorespiratory capacity (Dencker et al., 2008), but W_max_ is not only a measure of peak aerobic power but also anaerobic capacity and the ability to utilize higher threshold motor units (Rowland, 2017). However, only a few studies have investigated the associations of the measures of CRF, such as V̇O_2peak_ or W_max_ scaled by LM with cognition and have reported statistically insignificant relationships between CRF scaled by LM and cognition in youth (Haapala et al., 2015; Haapala, Lintu, et al., 2019; Raine et al., 2017, 2018).

Maximal indices of CRF, such as VO_2peak_ and W_max_, reflect only one aspect of CRF and different submaximal indices of CRF have been introduced. V̇O_2_ at the ventilatory threshold (VT), referring V̇O_2_ at the point where the rise in minute ventilation exceeds the increase in V̇O_2_ during the incremental exercise test, is one commonly used and non‐invasive measure of submaximal exercise capacity. VT has also been found to be more sensitive to changes in physical activity than V̇O_2peak_ in youth (Balady et al., 2010; Stringer, 2010). Furthermore, while the cardiac output is the strongest determinant of V̇O_2peak_, muscle oxidative capacity and potentially reduced blood supply determine VT (Wasserman, 1987). In addition, VT, reflecting the ability to sustain submaximal exercise for prolonged periods (Basset & Howley, 2000) could be an ecologically more valid measure of CRF than V̇O_2peak_ because habitual physical activity in youth is often light to moderate intensity and therefore rarely intensive enough to improve or require high V̇O_2peak_ (Armstrong, Tomkinson, & Ekelund, 2011). Because of these physiological differences, the associations of V̇O_2peak_ and VT with cognition and the mechanisms explaining those associations may differ. However, to the best of our knowledge, there are no previous studies that investigate the relation between VT and cognition in youth.

Body mass index (BMI) and waist circumference have been inversely associated with working memory, attention, psychomotor ability, and mental flexibility in youth (Bugge et al., 2018; Yau, Kang, Javier, & Convit, 2014). Similarly, higher body fat percentage (BF%) (Haapala et al., 2015; Kamijo et al., 2012) and visceral adipose tissue (Raine et al., 2017, 2018) have been inversely related to cognition tests in children. However, the inverse association between adiposity and cognition is not confirmed in all studies (Gunstad et al., 2008; Haapala, Lintu, et al., 2019). Although overweight and obesity are strongly related to arterial stiffness in youth and the associations between vascular health and cognition may already be present during childhood and adolescence (Lamballais et al., 2018; Vogrin et al., 2017), most previous studies have focused on middle‐aged and elderly adults (Lamballais et al., 2018).

Inappropriate scaling of CRF may limit our understanding on the associations of CRF with cognition. Furthermore, only a few studies have investigated the associations of CRF, adiposity, and arterial stiffness with cognition in adolescents simultaneously. We, therefore, investigated the relationships of V̇O_2peak_ and VT scaled by LM, BF%, and arterial stiffness with cognitive function in 16‐ to 19‐year‐old adolescents.

## 2 METHODS

### 2.1 Study design, participants, and laboratory procedure

The present analyses are based on the baseline data collected in the Neural Effects of Exercise, Diet, and Sleep (NEEDS) Study (ISRCTN12991197) in 2016–2017. Altogether, fifty‐four 16‐ to 19‐year‐old adolescents (19 males and 35 females) were recruited from high schools and vocational schools located in the city of Jyväskylä, Finland. The adolescents were eligible to participate in the study if they were apparently healthy. Exclusion criteria included any cardiovascular disease, untreated or poorly controlled type 1 diabetes, musculoskeletal trauma or disorder, or severe depression or anxiety. The protocol of the NEEDS Study was approved by the ethics committee of the University of Jyväskylä, Finland. All participants gave their written informed consent.

### 2.2 Assessments of body composition

BM, fat mass, BF%, and LM were measured twice by bioelectrical impedance analysis by the InBody 720 device (Biospace Co. Ltd., Seoul, South Korea) following a fast of at least 3‐hr (Stenman, Pesola, Laukkanen, & Haapala, 2017). In the present study, coefficients of variations for fat mass and LM analyses were 2.3 and 1.0%, respectively. Body composition assessed by bioelectrical impedance has been found to have an acceptable agreement with the measures of body composition measured by the dual‐energy x‐ray absorptiometry in children and adults (Sillanpää et al., 2014; Tompuri et al., 2015). Stature was measured twice in the Frankfurt plane without shoes by a wall‐mounted stadiometer with an accuracy of 1 mm. Waist circumference was measured twice after expiration at mid‐distance between the bottom of the rib cage and the top of the iliac crest using non‐stretchable measuring tape with an accuracy of 1 mm. The mean of these two values was used in the analyses. BMI was calculated as BM (kg)/stature (m)^2^ and ISO‐BMI was computed based on Finnish reference values (Saari et al., 2011). ISO‐BMI transforms age‐ and sex‐specific child BMI to corresponding adult BMI value. The prevalence of underweight (ISO‐BMI/BMI<17), normal weight (17–24.9), overweight (25–29.9), and obesity (≥30) was computed using standard cut‐offs.

### 2.3 Assessments of cardiorespiratory fitness

CRF was assessed by a maximal ramp exercise test on an electromagnetically braked Monark 929E cycle ergometer (Monark Exercise Ab, Sweden). The protocol included a 2‐min resting period seated on an ergometer, a 2‐min warm‐up without resistance (0 W), and an incremental exercise period with an increase of workload by 1 W/3 s (totaling 20 W/min) until voluntary exhaustion. The participants were asked to keep the cadence of 70–80 during the test. The test was terminated when the participant was unable to keep the cadence of 50 revolutions per minute or requested to stop. Participants were verbally encouraged to exercise until voluntary exhaustion.

Respiratory gas exchange was assessed directly by the breath‐by‐breath method using a metabolic cart (Vmax Encore, VIASYS Ltd., Conshohocken, USA) from the 2‐min resting period until the point of voluntary exhaustion and was averaged over 20‐s periods. The metabolic cart was calibrated according to the manufacturer's instructions. V̇O_2peak_ was defined as the highest V̇O_2_ achieved in the exercise test averaged over 20 s recorded during the last minute of the exercise test. The beat‐by‐beat heart rate (HR) during the exercise test was recorded using a Polar H7 HR sensor (Polar Electro, Kempele, Finland).

Because we did not perform a supramaximal validation test to obtain maximal oxygen uptake (Sansum et al., 2019), we considered exercise test maximal if the primary and secondary objectives and subjective criteria indicated maximal effort and maximal cardiorespiratory capacity (a plateau of V̇O_2_ regardless of increasing workload, HR >85% of predicted (Machado & Denadai, 2011), respiratory exchange ratio >1.05, or perceived exertion in Borg 6–20 scale ≥18, flushing, and sweating), and the exercise physiologist supervising the exercise test considered the test maximal.

V̇O_2peak_ was defined as mL × kg LM^−1^ × min^−1^, because V̇O_2peak_ scaled for mL × kg LM^−1^ × min^−1^ has been considered the most appropriate body size normalizing factor (Graves et al., 2013; Loftin et al., 2016). V̇O_2peak_ mL × kg LM^−1^ × min^−1^ was not statistically significantly associated with LM (r = 0.053, *p* = .702). V̇O_2_ at VT was determined by one experienced assessor (EAH) using the equivalents for V̇_E_/V̇CO_2_ and V̇_E_/V̇O_2_. V̇O_2_ at VT was defined as a rate of V̇O_2_ where V̇_E_/V̇O_2_ begins to increase without an increase in V̇_E_/V̇CO_2_. VT was confirmed using the V‐slope method. VT was defined as V̇O_2_ mL × kg LM^−1^ × min^−1^ and V̇O_2_ at VT as a percentage of V̇O_2peak_. VT scaled by LM was not associated with LM (r = 0.066, *p* = .636). Peak power output (W_max_) was defined as the maximal workload achieved at the end of the maximal exercise test and was scaled by LM. W_max_ scaled by LM was not statistically significantly associated with LM (r = −0.126, *p* = .365). To allow comparison with previous studies, we also performed the analyses using V̇O_2peak_, W_max_, and VT scaled by BM^−1^. V̇O_2peak_ and VT scaled by BM were inversely, but not statistically significantly, associated with BM (r = −0.219, *p* = .111 and r = −0.077, *p* = .580, respectively). W_max_ scaled by BM was inversely associated with BM (r = −0.302, *p* = .026).

### 2.4 Assessments of aortic wave velocity and augmentation index

Aortic pulse wave velocity (PWVao) and augmentation index (AIx%) were measured twice from the right upper arm in the supine position at about 2‐min intervals by oscillometric pulse wave analysis (Arteriograph; TensioMed Ltd., Budapest, Hungary) after a 10‐min rest in the supine position (ArterioGraph. Medexpert Ltd., 2010; Haapala, Laukkanen, Takken, Kujala, & Finni, 2018). The mean of these two measurements was used in the analyses. Initially, the Arteriograph automatically measured the actual systolic blood pressure (SBP) before inflating the cuff to a suprasystolic level of 35 mmHg above resting SBP and measured the fluctuations in the brachial artery. The device automatically measures resting SBP and diastolic blood pressure (DBP). The data were relayed to a tablet computer, recorded, and analyzed as pulse waves as described previously (Haapala, Veijalainen, Kujala, & Finni, 2019a). PWVao has been found to have good short‐term reproducibility and AIx modest short‐term reproducibility in youth (Haapala, Veijalainen, et al., 2019). Previous studies in adults have demonstrated a strong correlation (r > 0.9) between PWVao and central blood pressure assessed by the Arteriograph and invasively measured PWV (Horváth et al., 2010; Rossen et al., 2014). Furthermore, PWV and AIx assessed by Arteriograph device correspond relatively well with PWV and AIx measured by other commonly used non‐invasive techniques (Jatoi, Mahmud, Bennett, & Feely, 2009).

### 2.5 Assessment of cognition

Cognitive functions were assessed using a computer‐based CogState test battery (CogState Ltd, Melbourne, Australia) consisting of eight separate tests measuring accuracy, the speed of performance, executive functions, attention, visual learning and memory, and working memory. The CogState has been reported to be a valid tool to assess neurocognitive functions/functioning in adolescents (Maruff et al., 2009) and the tasks are not influenced by verbal skills or cultural background (CogState, 2019).

In the current study, we examined the eight main variables associated with the cognitive tests, which sub‐variables were related to the speed of the performance, accuracy or the total number of errors. All sub‐tests were performed on the computer consecutively in a pre‐determined order. All the cognitive tests of this study were performed in the same testing laboratory and on the same computer (Lenovo ThinkPad E550) in a peaceful and quiet room before the exercise test. Participants were provided with test presentations and instructions were reported according to the manufacturer's guidelines.

Accuracy during the working memory task was measured by Two Back Task (TWOB) and speed during short term memory task with One Back Task (ONB). In the TWOB, the participants were asked (using buttons “yes” or “no”) to answer whether a present card was the same as the card presented two cards ago. In the ONB the participants were asked, using buttons “yes” or “no” to answer whether the present card was the same as the previous card. A higher score indicated better performance.

Accuracy during the visual memory task was assessed with the One Card Learning test (OCL). In the OCL, the participants were asked (using buttons “yes” or “no”) to answer whether they had seen the present card before during the OCL test. Accuracy was scored as the reaction time required to make a correct response. A higher score indicated better performance.

Visual learning and memory (paired‐associate learning) were measured by the Continuous Paired Associate Learning Task (CPAL). In the first part of the two‐part test, the participants were asked to learn and remember abstract pictures and hidden patterns in different locations. Then the participants were then asked to recall where the re‐displayed hidden picture had been located. The test score was the errors during all performance sequences. A lower score indicated better performance.

Speed of performance during an attention task was measured by the Identification Task (IDN). During the task, the participants were asked to choose whether a revealed card was either red or not. If the card was red, the participants were required to press “yes” and for the black card to press “no.” A lower score indicated better performance.

Psychomotor function and reaction time were assessed with the Detection Test (DET). During the test, the participants were asked to click a button as quickly as possible when a playing card flipped over on a computer screen. The test result was based on accuracy, speed of performance, and the number of correct answers. A lower score indicated better performance.

Execution functions were assessed with the Groton Maze Learning Test (GML). Initially, the participants were asked to search hidden a 28‐step path on a computer screen and then remember the path as perfectly as possible. The visual memory was measured by Groton Maze Learning Test ‐Delayed Recall (GMR) which corresponds to the GML‐test.

### 2.6 Other assessments

Pubertal status was assessed by self‐reported testicular development in boys and breast development in girls on the basis of the five‐stage criteria described by Tanner (Tanner, 1949; Taylor et al., 2001).

### 2.7 Statistical methods

All statistical analyses were performed using IBM SPSS Statistics 24.0. for Macintosh (IBM Comp. Armonk, NY, USA). Differences in basic characteristics, V̇O_2peak_, V̇O_2_ at VT, body size and composition, PWVao, and Aix% between sexes were analyzed by the Student's *t* test or Mann–Whitney *U*‐test for continuous variables. Associations of CRF, body composition, and arterial stiffness (Aix% and PWVao) were compared to different cognition test variables using linear regression analyses adjusted for age and sex. Age and sex were entered into the regression model at the 1st step and the measures of CRF, body composition, or arterial health were entered separately into the model at step 2 and the standardized regression coefficients with corresponding *p*‐values were reported for each factor. Pubertal status was not included as a covariate into the regression models because we found that pubertal status was not associated with the measures of CRF or cognition. The *p*‐values <.05 were considered statistically significant.

## 3 RESULTS

### 3.1 Basic characteristics

Males were taller and heavier and had a lower BF% and a higher LM than females (Table 1). Males also had a higher absolute and body mass proportional to V̇O_2peak_ and W_max_ (*p* < .001) than females but no statistically significant differences were observed in V̇O_2peak_ and W_max_ scaled by LM between females and males. In addition, males had a higher V̇O_2_ at V̇T scaled by BM than females but there were no differences in V̇O_2_ scaled by LM between males and females. Males also had higher systolic blood pressure and lower AIx% than females. Males had a poorer performance in TWOB than females (*p* = .003), but there were no other statistically significant differences in cognitive performance between males and females.

**Table 1:** Characteristics of participants

	All (*N* = 54)	Girls (*n* = 35)	Boys (*n* = 19)	*p*
Age (years)^a^	17.0 (2)	16.9 (2)	17.1 (2)	.585
Weight (kg)	65.3 ± 9.7	62.3 ± 8.7	70.7 ± 9.3	.003
Stature (cm)	171.2 ± 7.6	167.0 ± 0.1	178,0 ± 0.1	<.001
ISO‐BMI	22.4 ± 2.9	22.4 ± 3.0	22.5 ± 2.7	.883
Weight status (*n*/ %)				.270
Underweight	1 (1.9)	1 (2.9)	0 (0.0)	
Normal weight	44 (81.5)	30 (85.7)	14 (73.7)	
Overweight	8 (14.8)	3 (8.6)	5 (26.3)	
Obese	1 (1.9)	1 (2.9)	0 (0.0)	
Pubertal status (%) (Tanner)				.575
Stage 1‐II	5.5	2,9	10.5	
Stage III	13.0	14.3	10.6	
Stage IV	31.5	28.6	36.8	
Stage V	50.0	54.3	42.1	
Waist circumference (cm)	74.6 ± 7.1	72.8 ± 6.4	78.1 ± 7.0	.004
Body fat mass (kg)	13.7 ± 6.6	15.9 ± 6.1	9.6 ± 5.6	<.001
Lean body mass (LM)(kg)	28.7 ± 5.2	25.5 ± 2.6	34.7 ± 3.2	<.001
Body fat percentage (%)	20.8 ± 8.5	25.0 ± 6.2	12.9 ± 6.2	<.001
V̇O_2peak_ (ml x min^−1^)	3,037 ± 690	2,655 ± 433	3,741 ± 492	<.001
V̇O_2peak_/lean body mass (ml/kg/min^−1^)	105.6 ± 13.6	104.0 ± 12.6	108.5 ± 15.2	.228
V̇O_2peak_/body weight (ml/kg/min^−1^)	46.7 ± 9.7	43.1 ± 7.2	53.8 ± 10.0	<.001
Ventilatory threshold 1 (ml/min^−1^)	1963 ± 565	1718 ± 397	2,415 ± 554	.004
VT/lean body mass (ml/kg/min^−1^)	68.17 ± 14.2	67.3 ± 13.1	69.7 ± 14.9	.497
VT/body weight (ml/kg/min^−1^)	30.2 ± 7.78	27.9± 6.7	34.4 ± 8.0	.004
V̇O_2_ at VT as % from V̇O_2peak_	64.7 ± 10.6	64.8 ± 10.6	64.4 ± 11.3	.821
Peak respiratory exchange ratio	1.28 ± 0.1	1.32 ± 0.8	1.21 ± 0.8	< .001
Resting heart rate (beats/min)	62 ± 8	64 ± 8	59 ± 8	< .001
Maximal heart rate (beats/min)	190 ± 8	189 ± 8	191 ± 8	.025
W_max_/body weight (ml/kg/min)	3.7 ± 0.7	3.4 ± 0.6	4.2 ± 0.6	< .001
W_max_/LM (ml/kg/min)	8.4 ± 0.9	8.3 ± 2.0	8.5 ± 0.8	.532
Resting systolic blood pressure (mmHg)	118 ± 9.9	114 ± 0.1	124 ± 8.5	.001
Resting diastolic blood pressure (mmHg)	66 ± 6.9	67 ± 6.9	65 ± 7.3	.531
Mean of Aortic PWV (m/s)	6.2 ± 0.7	6.2 ± 0.6	6.4 ± 0.9	.409
Mean of Augmentation index (%)	8.9 ± 7.2	10.5 ± 7.0	6.1 ± 6.7	.010

Note

LM = Lean mass, V̇O_2peak_ = Peak oxygen uptake, (the highest value achieved in the exercise test averaged over 20s recorded during the last minute of the exercise test), VT = ventilatory threshold, Ventilatory threshold 1 (ml/min^‐1^) = estimated V‐slope, V̇O_2_/VT/V̇O_2peak_ (%) = percentage of VT from V̇O_2peak_, W_max_ = maximal workload, PWV = Pulse Wave Velocity.

Data are from Student´s *t* test or Mann–Whitney *U*‐test for continuous variables. The data are displayed as means (±SDs) for normally distributed variables, ^a^medians (IQRs) for skewed distributions, or percentages (%). *p*‐values refer to statistical significance for differences between boys and girls.

### 3.2 Associations of cardiorespiratory fitness, body composition, and arterial stiffness with cognition

Higher V̇O_2peak_/LM was associated with better accuracy of working memory (TWOB) after adjustment for age and sex. Higher W_max_/LM was also associated with better accuracy of working memory (TWOB) and accuracy of visual learning (OCL) after adjustment for age and sex (Table 2). Furthermore, higher V̇O_2_ at VT/LM was associated with better working memory (TWOB), visual learning (OCL), as well as visual learning and memory (CPAL). V̇O_2_ at VT as a proportion of V̇O_2peak_ (%**)** was not associated with cognition. The associations of V̇O_2peak_, VT, and W_max_ scaled by BM with cognition are present in Table S1. The results based on the measures of CRF scaled by BM were relatively similar to the results based on the measures of CRF scaled by LM.

**Table 2:** The associations of peak oxygen uptake (V̇O_2peak_), W_max_, and the ventilatory threshold (VT) with Cogstate test scores

	TWOB (accuracy)		ONB (SoP)		OCL (accuracy)		CPAL (errors)		IDN (SoP)		DET (SoP)		GMR (errors)		GML (errors)	
	β	*p*	β	*p*	β	*p*	β	*p*	β	*p*	β	*p*	β	*p*	β	*p*
V̇O_2peak_/LM	0.356	**.011**	−0.079	.620	0.297	.057	−0.178	.232	−0.132,	.388	−0.038	.812	−0.167	.287	−0.082	.604
W_max_/LM	0.304	**.020**	0.022	.881	0.370	**.009**	−0.190	.165	−0.105	.453	0.039	.794	−0.169	.242	0.043	.769
VT/LM	0.302	**.016**	−0.125	.378	0.307	**.026**	−0.268	**.040**	−0.019	.887	0.012	.934	−0.250	.060	−0.230	.099
V̇O_2_ at VT as % from V̇O_2peak_	0.170	.207	−0.126	.399	0.224	.127	−0.242	.078	0.057	.695	0.068	.650	−0.209	.150	−0.245	.094

Note

The data are standardized regression coefficients with corresponding p‐values for each factor from linear regression analyses adjusted for age and sex. Statistically significant associations are bolded.

Abbreviations: CPAL, Continous paired‐associate learning test; DET, Detection test; GML, Groton maze learning test; GMR, Groton maze learning test (delayed recall); IDN, Identification test; LM, Lean body mass; OCL, One card learning test; ONB, One back test; SoP, Speed of performance; TWOB, Two back test; W_max_, peak power output

BF% and waist circumference were not associated with the measures of cognition after adjustment for age and sex (Table 3). We also tested the associations between ISO‐BMI and cognition, but ISO‐BMI was not associated with cognition (β = −0.228 to 0.091, *p* > .300). Moreover, AIx%, and PWVao were not associated with cognition (Table 3).

**Table 3:** The associations of waist circumference, body fat percentage, pulse wave velocity, and augmentation index with Cogstate test scores

	TWOB (accuracy)		ONB (SoP)		OCL (accuracy)		CPAL (errors)		IDN (SoP)		DET (SoP)		GMR (errors)		GML (errors)	
	β	*p*	β	*p*	β	*p*	β	*p*	β	*p*	β	*p*	β	*p*	β	*p*
Waist circumference (cm)	0.018	.904	0.053	.745	−0.107	.506	−0.013	.931	−0.105	.492	−0.033	.839	0.020	.900	−0.092	.569
Body fat percentage (%)	−0.120	.542	0.141	.518	−0.242	.262	0.098	.631	0.023	.913	−0.272	.202	−0.160	.458	−0.045	.835
Pulse wave velocity (m/s)	0.019	.888	0.097	.509	−0.127	.384	0.192	.157	0.026	.855	−0.110	.453	−0.019	.896	−0.071	.627
Augmentation index (%)	0.092	.501	0.135	.369	−0.001	.992	−0.028	.841	−0.081	.576	0.125	.406	−0.110	.455	0.054	.720

Note

The data are standardized regression coefficients with corresponding p‐values for each factor from linear regression analyses adjusted for age and sex.

Abbreviations: CPAL, Continous paired‐associate learning test; DET, Detection test; GML, Groton maze learning test; GMR, Groton maze learning test (delayed recall); IDN, Identification test; OCL, One card learning test; ONB, One back test; SoP, Speed of performance; TWOB, Two back test

## 4 DISCUSSIONS

We observed that higher V̇O_2peak_, V̇O_2_ at VT, and W_max_ were associated with better working memory accuracy. V̇O_2_ at VT and W_max_ were also positively associated with visual learning. Finally, we found no statistically significant association of BF%, waist circumference, AIx%, or PWVao with cognition.

In line with previous studies (Chaddock, Erickson, Prakash, Kim, et al., 2010; Chaddock et al., 2011; Raine et al., 2013), we found a positive association between VO_2peak_ and working memory, while W_max_ was also positively associated with working memory. W_max_ had a stronger association with visual learning than VO_2peak_, which is in contrast with some previous studies suggesting no association between W_max_ and cognition in children (Haapala et al., 2015; Haapala, Lintu, et al., 2019). Furthermore, we found no associations between VO_2peak_ or W_max_ with executive function inconsistent with the findings of others (Davis et al., 2011; Wu & Hillman, 2013). The reason for a larger and statistically significant regression coefficient in the association of W_max_ with visual learning compared to that of V̇O_2peak_ is not known, but one explanation might be that W_max_ also reflects neuromuscular functioning and coordination, which have been associated with better cognitive functions in children and adults (Geertsen et al., 2016; Haapala et al., 2015). In addition, while a previous study (Raine et al., 2017) has suggested that the associations between CRF and cognition might reflect adiposity rather than peak aerobic power, our findings suggest that higher VO_2peak_ and W_max_ are appropriately scaled by LM benefits working memory and visual learning in adolescents. These findings also agree with some previous studies showing a positive association of fitness with more complex and higher order cognition functions, but not with the performance in simple reaction speed tasks (Hillman, Erikson, & Kramer, 2008). However, direct comparison between the present study and previous studies is unfortunately not possible because of the different measures of cognition and approaches to scale VO_2peak_. Furthermore, our results using the measures of CRF scaled by LM were remarkably similar to the results utilizing the measures of CRF scaled by BM. One explanation for these similar results may be that our study sample was relatively homogenous, muscular, and fit. Therefore, for example, VO_2peak_ scaled by BM was not statistically significantly associated with BM, indicating that VO_2peak_ scaled by BM was not heavily confounded by body size and composition. More studies with larger and more heterogenous samples comparing different scaling approaches for CRF are warranted.

Better CRF has been associated with improved brain synaptic plasticity and grey matter development and white matter integrity in children and adolescents (Chaddock‐Heyman et al., 2014; Cotman, Berchtold, & Christie, 2007; Talukdar et al., 2018). Furthermore, CRF has been positively related to cerebral blood flow (Chaddock, Erickson, et al., 2012; Tyndall et al., 2018), exercise‐activated growth factors (Cotman et al., 2007; Hillman et al., 2008), and increased neural processing (Gomez‐Pinilla & Hillman, 2013). These brain level structural and functional changes may explain the associations between CRF and cognition. It is also possible that improved V̇O_2peak_ and W_max_ due to regular vigorous physical activity partly may explain our results (Talukdar et al., 2018).

To the best of our knowledge, there are no previous studies on the associations between V̇O_2_ at VT and cognition in adolescents. We observed positive associations of VT with working memory and visual learning. Higher VT refers to the point at which lactate production exceeds lactate clearance because of the increased contribution of anaerobic metabolism (Svedahl & MacIntosh, 2003). In addition, VT is limited by muscle oxidative capacity and possibly through reduced blood supply is limiting VT (Wasserman, 1987). The mechanism for how higher VT may benefit cognitive functions is currently unknown. It is possible that physical activity partly explains observed associations as VT has been found to be more sensitive to exercise training than V̇O_2max_ (Balady et al., 2010; Stringer, 2010). Furthermore, VT has been inversely associated with arterial stiffness in adolescents (Haapala et al., 2018) and one plausible mechanism explaining our observations may be that higher VT influences cognition through better cerebral vascular functions and thereby improved oxygen and nutrient supply (Wasserman, 1987). Nevertheless, biological brain level mechanisms explaining the associations of CRF, VT, and arterial health with cognitive functions are not well understood and further studies are needed.

While unfavorable cardiometabolic risk factor profile has been linked to poorer cognition in youth, we found weak if any associations of adiposity and arterial stiffness with cognition. Our results are in line with some previous studies showing a weak and statistically non‐significant association between adiposity and cognition (Gunstad et al., 2008). Nevertheless, other studies have shown poorer cognition in overweight and obese children than in their normal‐weight peers (Yau et al., 2014). Similarly, arterial stiffness has been related to impaired cognitive function in the elderly (Kramer et al., 2006). Furthermore, higher Aix(%) and central systolic artery pressure have been associated with lower academic performance among youth aged 11–16 years (Vogrin et al., 2017), but the evidence in youth and young adults is limited. The reason for the weak associations of adiposity and arterial stiffness with cognition in the present study might be that the adolescents in our study were relatively lean and most of them were normal weight and had normal levels of arterial stiffness.

The strengths of the present study are valid and objective measures of cognition, CRF, arterial stiffness, and body composition. We also studied the associations of CRF, adiposity, and arterial stiffness with cognition in adolescents, which is an understudied population. Nevertheless, we did not perform the supramaximal validation test to confirm maximal oxygen uptake and therefore it is possible that we have underestimated true aerobic capacity in some participants (Sansum et al., 2019). Furthermore, we had a relatively small sample size and unequal sex distribution. Therefore, we were not able to perform the analyses separately for males and females and could not stratify the analyses based on pubertal development. Furthermore, we did not control for socioeconomic status in the analyses, because our sample was a very homogeneous group. The sample was also recruited from local high‐schools and therefore the sample may not be representative of the general population of adolescents from varying socioeconomic backgrounds. Finally, our cross‐sectional design precludes any causal interpretations.

In conclusion, adolescents with higher CRF and especially those with higher VT, performed better in tasks requiring working memory and visual learning. Furthermore, adiposity and arterial stiffness were not associated with cognition. In the future, more research is warranted to explore whether V̇O_2peak_ or VT in adolescence predicts brain health and cognitive functions later in life.

## CONFLICTS OF INTEREST

The authors declare no conflict of interest and have no financial relationship to disclose.

## AUTHOR CONTRIBUTIONS

Conceptualization; EAH, HS, HLH, and NL; methodology and data collection: HS and EAH; writing—original draft preparation: HS; writing—review and editing: NL, HLH, and EAH. All authors have read and agreed to the submitted version of the manuscript.

## Supporting information



Table S1
