# Supplementary material for: Associations of cardiorespiratory fitness, adiposity, and arterial stiffness with cognition in youth
Source: Physiol Rep. 2020 Sep 19;8(18):e14586. doi: 10.14814/phy2.14586 (PMC7507089; doi:10.14814/phy2.14586)
Supplement: Supplementary file 1 — Table S1 [file PHY2-8-e14586-s001.docx]

**Supplementary table 1.** The associations of peak oxygen uptake (V̇O_2peak_), W_max,_ and ventilatory threshold (VT) scaled by body mass with Cogstate test scores.

|  | TWOB  (accuracy) |  | ONB  (SoP) |  | OCL (accuracy) |  | CPAL  (errors) |  | IDN  (SoP) |  | DET  (SoP) |  | GMR  (errors) |  | GML  (errors) |  |
| --- | --- | --- | --- | --- | --- | --- | --- | --- | --- | --- | --- | --- | --- | --- | --- | --- |
|  | β | p | β | p | β | p | β | p | β | p | β | p | β | p | β | p |
| V̇O_2peak_/BM | 0.368 | **0.031** | -0.172 | 0.375 | 0.374 | **0.047** | -0.196 | 0.275 | -0.156 | 0.398 | -0.162 | 0.398 | -0.084 | 0.567 | -0.035 | 0.856 |
| W_max_/BM | 0.329 | **0.040** | -0.060 | 0.738 | 0.425 | **0.015** | -0.204 | 0.222 | -0.128 | 0.458 | -0.068 | 0.703 | -0.100 | 0.573 | 0.092 | 0.606 |
| VT/BM | 0.324 | **0.020** | -0.170 | 0.277 | 0.333 | **0.029** | -0.299 | **0.038** | -0.042 | 0.783 | -0.066 | 0.674 | -0.226 | 0.140 | -0.221 | 0.153 |

The data are standardized regression coefficients with corresponding p-values for each factor from linear regression analyses adjusted for age and sex. Statistically significant associations are bolded.

BM= Body mass, W_max_= peak power output, TWOB= Two back test, ONB= One back test, OCL= One card learning test, CPAL= Continous paired associate learning test,

IDN= Identification test, DET= Detection test, GMR= Groton maze learning test (delayed recall), GML= Groton maze learning test, SoP= Speed of performance
